# Glutamate dehydrogenase: a novel candidate to diagnose *Plasmodium falciparum* through rapid diagnostic test in blood specimen from fever patients

**DOI:** 10.1038/s41598-020-62850-x

**Published:** 2020-04-14

**Authors:** Lokesh D. Kori, Neena Valecha, Anupkumar R. Anvikar

**Affiliations:** 1 ICMR-National Institute of Malaria Research, Dwarka-8, New Delhi, 110077 India; 2 Former Director, ICMR-National Institute of Malaria Research, Dwarka-8, New Delhi, 110077 India

**Keywords:** Microbiology, Infectious-disease diagnostics, Malaria

## Abstract

In recent years, *Plasmodium falciparum* histidine-rich protein 2 gene deletion has been reported in India. Such isolates are prone to selective transmission and thus form a challenge to case management. As most of the rapid malaria diagnostic tests are based on the detection of HRP2 protein in the blood, we attempted to use Glutamate Dehydrogenase (GDH) as a biomarker for the diagnosis of *P. falciparum*. Recombinant PfGDH was successfully cloned, expressed and purified using the Ni-NTA approach. Polyclonal antibodies were raised against full-length rPfGDH and its peptides. Antibodies for rPfGDH showed a strong immune response against the recombinant protein. However, antibody showed no affinity towards the peptides, which suggests they failed as antigen. Antibodies for rPfGDH significantly detected the GDH in human blood specimens. This is the first report where *P. falciparum* GDH was detected in malaria cases from various parts of India. The raised polyclonal antibodies had shown an affinity for PfGDH in quantitative ELISA and are capable to be exploited for RDTs. This research needs further statistical validation on a large number and different sample types from candidates infected with *P. falciparum* and other species.

## Introduction

In the era of malaria elimination, several African and South East Asian countries are pushing their limits to be free from malaria. However, these countries are facing the challenges related with malaria diagnosis. In South East Asia region, India accounts for about 80% of malaria cases and 60% deaths due to malaria^1^. For malaria diagnosis, different invasive methods are broadly in use. Slide microscopy remains the gold standard to identify the parasite and their load in malaria patient. Molecular biology based approaches are highly sensitive and accurate but are not suitable for rural areas due to high cost, complex methodologies, expensive equipments and skilled manpower. However, the invasive methods always have concerns such as fear of contacting with other blood related diseases, pain associated with finger pricking, proper disposal of needles, correct interpretation of results and adherence to hygiene practices^2,3^. In non-invasive approach saliva collection is feasible, to check the presence of parasitic biomarkers using rapid diagnosis test (RDT) and may serve as an excellent surveillance tool for malaria elimination programme^2^. Whole saliva samples from children with uncomplicated malaria showed 77.9% sensitivity against 97.9% from blood and 48.4% from supernatant of spun saliva samples, using lactate dehydrogenase (LDH) RDT and genotyping of *P. falciparum*^4^.

Antigen based RDT kits, available commercially lacks in the expected specificity and sensitivity (Table 1). In past few years, *hrp2* and *hrp*3 single or dual gene deletions were reported from Indian *P. falciparum* isolates and other parts of the world^5–7^. Bharti *et al*. has evaluated more than 20 brands of RDT based on HRP2 and their sensitivity varied between 80–97% for *P. falciparum*^8^. Deletion of *hrp2*/*3* gene in *P. falciparum* raises a concern for the national programme as HRP2 based RDT’s are broadly used for malaria diagnosis, this may result in misdiagnosis and false treatment strategies. A recent study showed that in eight malaria endemic Indian states *P. falciparum* Glutamate dehydrogenase (GDH) is genetically conserved and is under negative selection as observed by tajima D test^9^. GDH can be exploited as a potential marker for detection of *Plasmodium falciparum*.

**Table 1:** An overview of available biomarkers used to detect malaria.

Antigens	Properties	Remarks
Lactate dehydrogenase	-pLDH is specific for *P. falciparum and P. vivax*	Low sensitivity, need high parasitemia
Aldolase	-Aldolase based assays only confirms the presences of *Plasmodium* but cannot distinguish different species.	Not species specific, Cannot guide treatment
Histidine Rich Protein II (HRPII)	-HRP2 is highly sensitive assay.	Persistent positively of HRP2 tests after effective treatment. HRP2 gene deletion has been reported in past years, which urges urgent development of new biomarker candidate. HRP2 based assay is restricted to *Plasmodium falciparum*.
Glutamate dehydrogenase	-GDH is present in different isoforms in malaria parasite. -In *P. falciparum* 2 genes coding for GDH are present on chromosome 14 and 1 gene on chromosome 8. -Plasmodium GDH contain a unique N terminal residues and are found throughout the intraerythrocytic cycle of parasite. -Tertiary structure of PfGDH1 and PfGDH2 has been solved, which suggests that GDH need to be exploited as tool of malaria detection and drug target. -GDH is also present in other *Plasmodium* species.	GDH Epitope specific antibodies can used as Potential biomarker for Malaria detection. GDH is species specific.

Malaria parasites uses GDH as crucial enzyme to obtain energy via Krebs cycle, where it oxidizes glutamate to alpha-ketoglutarate utilizing NADP and releasing NADPH during intraerythrocytic stage^10^. GDH is approximately 50–60 kDa sized metabolic soluble protein^11,12^. In *P. falciparum* three *gdh* genes encoding potential GDH proteins are present, two genes are on chromosome 14 (PF14_0164 and PF14_0286; GDHa and GDHb) and one gene on chromosome 8 (PF08_0132; GDHc)^13,14^. Glutamate dehydrogenase was first isolated and characterized from the plasma of *Plasmodium falciparum* infected malaria patients^12^. GDH is a heat resistant and soluble antigen which can be used for antibodies production to improve immunodiagnostic assays^15,16^. Malaria parasite shows similar metabolism as host but the characteristics of the GDH enzyme are different based on their kinetical, electrophoretically, specificity of co-factors, substrates, degree of affinity, and immunogenicity. Reason behind the selection of Glutamate dehydrogenase is the exhibition of NADP-specific GDH activity. Malaria parasite secretes NADPH in combination with NADP-specific isocitrate dehydrogenase, which is not found in host red blood cells. Reports had shown that the GDH from a) animal origin requires purine nucleotide and b) *P. chabaudi* (rodent malaria parasite) does not require purine nucleotides^17^. GDH is one of the *P. falciparum* enzymatic antigen which has been immuno-detected by the antibodies raised in animals or from the sera of *P. falciparum* malaria patients from Yanumana Amerindians living in Venezuela^12^. Also, a recent study showed that Glutamate dehydrogenase gene sequence is highly conserved in *P. vivax* and used for diagnosis of vivax malaria in South Korea. Antibodies against PvGDH did not cross react with the sera from *P. falciparum* positive patients. Seol *et al*. suggested that GDH could be used as a potential antigen for seroepidemiology studies^18^. Therefore, we attempted to investigate *P. falciparum* GDH as a marker protein for malaria parasites^19,20^. In this article, PfGDH in blood sample from Indian malaria patients was investigated.

## Results

### Cloning, expression and purification of rPfGDH

The glutamate dehydrogenase gene (*pfgdh*) of *P. falciparum* 3D7 located on chromosome 14 was successfully expressed in BL21(DE3) *E. coli* cells. rPfGDH was purified more than 90% with Ni-IMAC and analyzed on SDS-PAGE and confirmed by Western Blot.

### Antibodies raised against rPfGDH and synthetic peptides

The antibody response against rPfGDH and synthetic peptides from ICR female mice was assayed by ELISA. Results were compared with the control sera obtained from the mice injected only with 1xPBS and adjuvant.

### Polyclonal antibodies response against PfGDH and peptides

Antibodies generated against rPfGDH protein and peptides were analysed using ELISA. Various concentrations of rPfGDH and peptides (1.25, 2.5, 5 and 10 μg) were used to check the response of the polyclonal antibodies. It was found that only antibodies against full length rPfGDH has shown affinity, wells with less concentration of protein has shown maximum binding. Results showed that none of the predicted PfGDH peptides are eligible for *P. falciparum* detection.

### Serological analysis by ELISA

**Figure 1:**
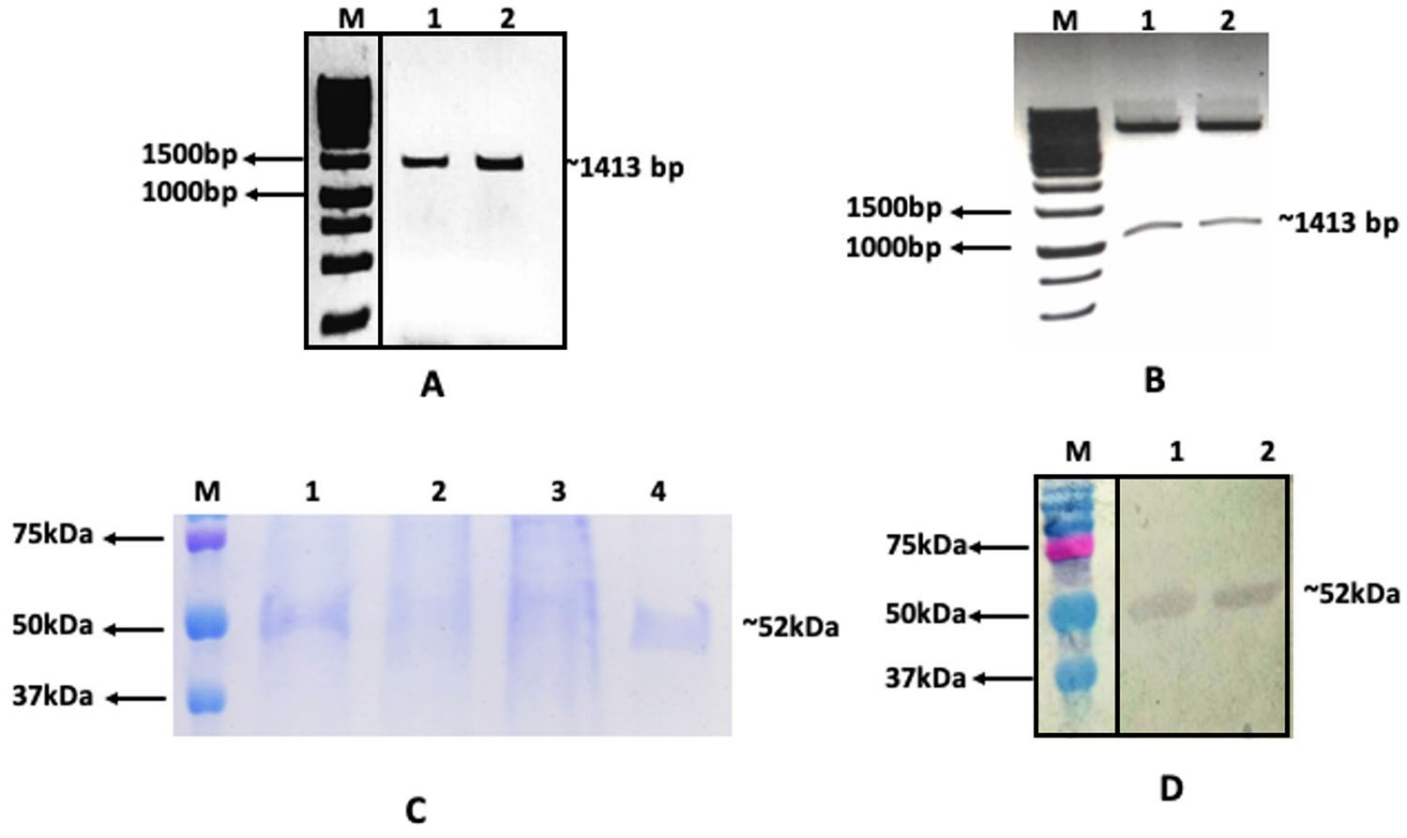
(**A**) **Gene amplification, Lane M- 1 Kb DNA ruler (Thermo scientific #SM0314), lane 1,2,3 rPfGDH gene amplification using gradient PCR at 57.9 °C, 56.1 °C and 55.0 °C. (**B**) Restriction enzyme digestion, Lane - C is intact pET22b, M - 1 Kb DNA ruler, 1 and 2 are digested clone with rPfGDH gene (~1413 bp). (**C**) Protein purification using Ni-IMAC gravity column, M. Dual colour protein Marker (Biorad), 1. PfGDH cell lysate with 1.5 M GuHCl, 2. Flow through, 3. Wash with 20 mM imidazole, 4. Wash with 50 mM imidazole, 5. Elution with 150 mM imidazole buffer. (**D**) **Western Blot of rPfGDH, lane M Dual colour protein Marker (Biorad), 1 and 2. rPfGDH samples eluted with 20 and 50 mM imidazole buffers. **Areas inside the black boxes are from the same gel respectively (given in supplementary file), they were cropped and linked together for clear visualisation and publication purpose only.

Quantitative ELISA was performed on the samples from DBS, blood pellet, serum and plasma. Total 41 samples (27 known positive and 14 known negative for *P. falciparum* and *P. vivax* validated by microscopy and PCR) were screened to detect the presence of Pf glutamate dehydrogenase by direct ELISA method. Out of twenty-four positive samples, 23 samples showed binding with the rPfGDH antibodies. On the other side, the negative samples did not show any significant response against the antibodies. All the experiments were performed in triplicate and were repeated to confirm the assay outcome. The cut-off value was the mean +2 SD of negative control (sample from uninfected human blood). The geometric mean of responses recorded from above samples showed significant outcome as the value of P < *0.0001*^21^ (Fig. 1).

### Diagnostic performance of anti-PfGDH antibodies

We have analysed total 27 anonymized known malaria samples, that included known positive *P. falciparum* samples WBC depleted RBC’s (n = 14) (upto 200 parasites/μl), dried blood spot (n = 5), plasma (n = 4) and serum samples (n = 4) and known negative specimens (n = 14) for *P. falciparum* (n = 8) and positive *P. vivax* (n = 6). The blood specimens with no *P. falciparum* (diagnosed with microscopy and PCR) were used as negative controls in Quality assurance laboratory at ICMR-NIMR, a WHO recognized facility.

We analysed anti-PfGDH antibodies for sensitivity and specificity using MedCalc statistical online software. The anti-PfGDH antibodies are 96.30% sensitive and 100% specific to PfGDH antigen, where the samples were true positive (n = 26), false negative (n = 1); true negative (n = 14) and false positive (n = 0).

### Comparison between study sample subgroups

All study sample subgroups were compared and shown in table no. 2 (supplementary data). Infected RBC OD value was found altered as compared to uninfected RBC OD values (p-value = 0.005). Similarly, DBS and plasma have shown altered OD values than uninfected RBC; p-value = 0.002 and p-value < 0.0001 respectively. Interestingly the OD value of infected RBC group was found significantly altered with plasma group (p-value = 0.018). Moreover, the OD values of infected RBC versus DBS groups and OD values of DBS versus plasma groups were not significantly altered (p-value > 0.05). The statistical analysis was performed using SPSS software version 17.

### Significance of antibodies against PfGDH in diagnosis

The presence of GDH antigen in blood specimen collected from malaria patients was detected (Fig. 1). A various sample subgroups were screened to check the sensitivity and specificity of the antibodies against PfGDH. The results demonstrate GDH as a soluble protein, as it was detected in plasma, serum, eluted DBS and RBC pellets.

## Discussion

The anti-GDH antibodies showed strong sensitivity (96.30%) and specificity (100%) towards PfGDH antigen (Fig. 1). The samples analysed were from the malaria endemic parts of the country and preserved at ICMR-NIMR. From ELISA results it was observed that the antigen-antibody affinity was slightly higher in plasma and WBC depleted RBC samples compared to the serum and eluted DBS specimens. This suggests that the whole blood specimens could be most appropriate sample type for carrying out the malaria diagnosis using anti-GDH antibodies.

For optimization of immunoassay, ELISA was repeatedly performed on the given specimens to precise the incubation period between the antigen and primary and secondary antibody. Similarly, for blocking 2.5% BSA was optimized and washing of wells were also refined. Assay optimization improved the cut-off value by reducing the background noise significantly.

The antibodies raised against the four predicted peptides of PfGDH have not responded during the immunoassay. These peptides may not be the suitable epitope to generate immunogenicity. As the sera did not show any response against the peptide, was not tested on the human specimens due to insufficient quantity.

To overcome the challenges associated with polyclonal antibodies (PAb) such as cross-reactivity against different epitopes, variability due to different animals and time, and to improve the sensitivity of the assay, attempts are in progress to develop monoclonal antibodies (MAb) against the recombinant PfGDH. The MAb will enhance the reproducibility, sensitivity and reduce the background noise. MAb are preferred over PAb to develop the assay into the RDT kits.

In recent years, increase in the prevalence of *hrp2* gene deletion has been observed^5–7^. HRP2/3 based RDT kits are commercially available and a key tool in malaria diagnosis before the treatment is given. The significant challenges associated with the currently available RDT kits are their maintenance and stability in fields which are remotely located and have very less or no resources to maintain the quality of these kits. These issues substantially affect the quality of the RDT results, which leads to misdiagnosis and implementation of wrong treatment or management of the disease.

To overcome *hrp2* gene deletion is a challenge and there is an urgent need to identify, develop and bring a new biomarker with the robustness to resist against the gene deletion in parasitic genome and overcome other issues. Since past few years, several researchers has been indicating towards Glutamate dehydrogenase protein which have potential to be developed as a biomarker to detect malaria parasite^22,23^. PfGDH protein encoded gene can stay tough in the field as it is present in three copy number on chromosome 8 and 14^13,14^. In a recent study from India, the authors found PfGDH as genetically conserved in eight highly malaria endemic states^9^. PfGDH is also heat resistant^15,16^ and could be exploited in RDT kits for real time field conditions, where logistics, temperature and humidity are the major challenges.

Ling *et al*.^24^ reported that polyclonal antibodies raised against *P. knowlesi* were reacted with NADP specific glutamate dehydrogenase of *P. knowlesi*, *P. falciparum* and *Proteus spp*. Similarly, monoclonal antibodies raised against GDH from *Proteus spp*. were cross-reacted with *P. falciparum*. Further GDH is known to be specific to malaria parasites^18,22^, this observation is concurrent with PfGDH antibodies which showed discrimination with *P. vivax* samples. This could be due to the distinct epitope region, other than substrate - enzyme binding site^22^. In this pilot study PfGDH was detected in samples with low parasitemia (upto 200 parasites/μl). Our findings are supported by the outcomes from a recent study where PvGDH was detected in samples with low (<1000) and high (>10000) parasitemia count without any significant difference^18^. This data potentiates the hypothesis of detecting the GDH antigen in soluble form in low parasitemic samples.

Blood sample subgroups comparison showed significant outcomes. Anti-rPfGDH antibodies showed strong response with the plasma, which confirms the presence of soluble GDH^11,18^. With respect to the infected RBC and eluted DBS samples no significant difference was observed, which suggests that both sample types are equally capable to detect the PfGDH antigen.

Due to non-availability of other *Plasmodium* species infected blood samples, we performed assay with *P. vivax* samples using anti-rPfGDH antibodies and interestingly there was no cross-reactivity observed. It was also noticed that anti-PfGDH antibodies show no cross-reaction with any of the human enzymes, similar observation was made by Rodriguez-Acosta *et al*. when they characterized the hyper immune sera against PfGDH from Yanumana Amerindians^12^ With these promising results, in future blood specimens infected with *P. vivax*, *P. malariae*, *P. ovale* and *P. knowlesi* from different endemic regions of India will be tested. We will attempt to perform the study on samples with low to high parasitemia to check to lower and higher limits of anti-PfGDH antibodies sensitivity. The outcome from those studies will help us to further optimize and strengthen the development of PfGDH antigen as biomarker for malaria diagnosis.

## Methods

### Ethics statement

The study was approved by the Institutional Human Ethics Committee (reference no. ECR/NIMR/EC/2017/64) to use already collected anonymized specimens with *P. falciparum* infection confirmed by microscopy and RDT in form of dried blood spot, whole blood, plasma and serum, preserved at ICMR-National Institute of Malaria Research, New Delhi, India.

To perform animal experiments, the study was approved by Institutional and Animal Ethics Committee (reference no. IAEC/NIMR/2018-2/10) following the national guidelines from The Committee for the Purpose of Control and Supervision of Experiments on Animals (CPCSEA Registration No. 33/GO/ReBi/S/99/CPCSEA), Government of India.

### Primer Design and PCR

**Figure 2:**
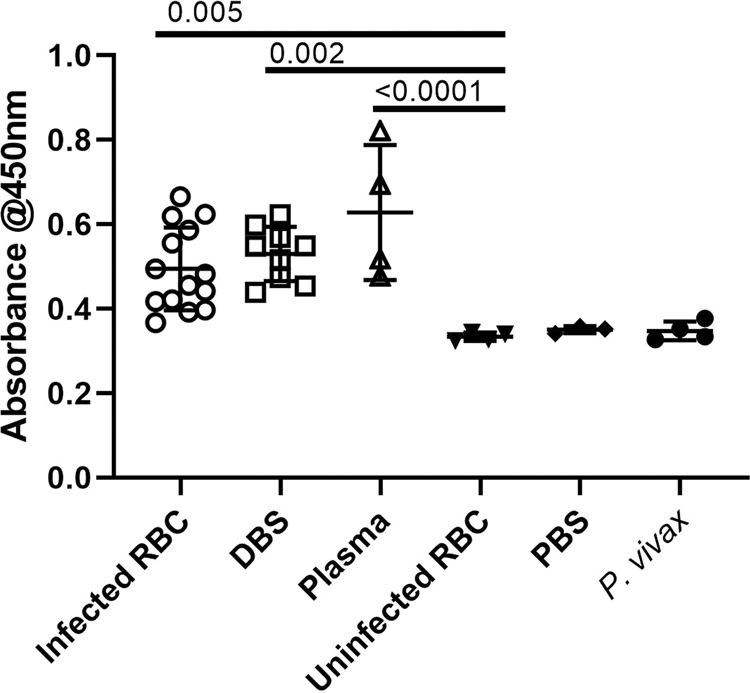
ELISA results showing the detection of *P. falciparum* GDH as protein biomarker by anti-PfGDH antibodies in a variety of blood samples, negative controls (uninfected RBC and PBS) and *P. vivax* infected RBC sample to check cross-reactivity.

The glutamate dehydrogenase gene (*pfgdh*) of 1413 bp (NCBI accession no. AY040586.1; encodes 470 amino acids) was PCR amplified using the forward primer 5′- CGGCATATGAGTGCTCTTAAAGACAAAACGG-3′ (the *NdeI* restriction enzyme site is underlined) and reverse primer 5′-CGGCTCGAG*TCAATGATGGTGATGGTGATG*ACAACCTTGTTC-3′ (the *XhoI* restriction site is underlined and the oligonucleotide expressing the 6xhistidine fusion peptide is italicized). Molecular recipe to amplify GDH gene included 0.25U of Q5 High Fidelity DNA polymerase, 1x Q5 reaction mixture and 1x Q5 GC content enhancer (NEB, England), 2 ng gDNA isolated from *Plasmodium falciparum* positive blood sample, 1 μM of each primer and 2 μM dNTP’s (GCC Biotech, India) in total 25 μl reaction volume. The thermal profile to amplify GDH gene was 98 °C-30 sec, followed by 30x cycle of 98 °C-7 sec, 55 °C-20sec, 72 °C-1 min and final elongation for 2 min at 72 °C, with 10 °C as storing condition. PCR amplified products were observed in 1% agarose gel run in 0.5x TBE buffer under UV transilluminator (Fig. 2A).

### Gene cloning

The amplified PCR product was digested using *NdeI* and *XhoI* restriction enzymes and the digested product was gel purified using a QIAquick gel-extraction kit (Qiagen, Germany) and cloned into the pET22b+ expression vector (Fig. 2B) (Novagen) using T4 DNA ligase enzyme (NEB, England) and transformed into DH5alpha *E. coli* competent cells to increase the copy number, subsequently the recombinant vector with *pfgdh* gene was transformed into BL21 (DE3) *E. coli* cells as per manufacturers recommended protocol. The clone designated with rPfGDH the expresses the protein of interest (residues 1–470 plus 6xHis tag at C-terminal) was selected for further studies.

### Protein expression and purification

The rPfGDH clone was grown at 37 °C in 1 l Terrific broth containing 0.5% glucose (Himedia, India) supplemented with 100 μg/ml ampicillin to an OD_650_ value of 0.4–0.6, was induced with 1 mM of Isopropyl β-D-1-thiogalactopyranoside (IPTG) and the culture was further incubated for 6 h at 37 °C at 225 rpm. Subsequently, the culture was centrifuged at 8000 g for 10 min to harvest the cells and stored at −20 °C. Later, cell pellet was resuspended in 20 ml GenePro Total Protein isolation buffer (Genetix, India) and incubated on ice for 10 min and centrifuged at 16000 g for 10 min. The clear cell lysate was collected and mixed with 1.5 M GuHCl and passed through 2 ml bed volume of cOmplete His-Tag purification resin (Roche, USA) using gravity flow columns (Biorad, USA). The column was washed with 4 bed volumes of buffer containing 40 Tris-HCl pH 7.4 and 20 mM and 50 mM Imidazole. rPfGDH was eluted with 100 and 150 mM imidazole, both eluates were mixed and passed through ultra-centrifugal device with a cut-off of 30 Kda (Ultracell, Merck, Millipore) at 5000 g for 10 min at 4 °C. The purification procedure was monitored by SDS-PAGE denaturing conditions, where the rPfGDH was appeared approximately as a ~52 kDa, which was in the agreement with its theoretical molecular mass (Fig. 2C). The rPfGDH was confirmed by his-tag monoclonal antibodies, mouse (Puregene, Genetix, India) and goat-anti mouse IgG-HRP antibodies on western blot using Biorad Mini-PROTEAN Tetracell system (Fig. 2D). The rPfGDH protein concentration estimated was 4 mg/ml against BSA standard.

### B-cell epitope prediction

Bepipred Linear Epitope Prediction an online tool (http://tools.iedb.org/bcell/) using hidden Markov model and propensity scale method was used to predict epitopes. The four dominant peptides considered for this study with high immunogenicity score, linear epitope region and hydrophilicity were: a) MGGGKGGSDFDPKGKSDN, b) PCTDVPAGDIGVGGR, c) NEQYSSDKYFPTFEET, d) PFQQGKLRKNGGVPHD. All the peptides were synthesized by GL Biochem Ltd, Shanghai, China.

### Polyclonal antibody generation

Polyclonal antibodies against rPfGDH and selected four above mentioned peptides were raised in ICR Swiss female mice (five weeks old), purchased from ICMR-National Animal Resource Facility for Biomedical Research, Hyderabad and kept at in-house animal facility of NIMR for the duration of experiments.

On day one each mouse (five mice per group) was immunized with 100 μg antigen (30 μl) (rPfGDH and peptides) mixed with equal volume of Complete Freund’s Adjuvant (Sigma, USA) at subcutaneous site using 30 g x5/16 U-40 syringe (Dispovan). Subsequently, two booster doses were given on day 21 and day 42 with 50 μg (30 μl) antigen in Incomplete Freund’s Adjuvant (Sigma, USA). Control mice were injected with 1xPBS and the respective adjuvant. On day 50, first bleed approximately 100 μl was collected from tail vein per mouse. All the animals were given with third and fourth booster doses on day 57 and day 71, second bleed was collected on day 79. From blood serum was separated after incubating the it at 37 °C for 30 min followed by a centrifugation at 2500 rpm for 5 min and stored at −20 °C for later use.

### Anti-PfGDH antibody purification

Polyclonal antibodies raised against full length PfGDH and above mentioned peptides were purified using Nab Spin Columns (Thermo Scientific, USA) by following the recommended protocol from the manufacturer. 100 μl of serum sample was mixed with the resin in the columns and was washed three times with binding buffer and subsequently eluted with 400 μl of elution buffer. Purified samples were aliquoted and stored at −20 °C for downstream experiments.

### Immune response test

rPfGDH and peptides (100 μl of a 1 μg/ml solution in 1xPBS) was coated in the wells of high binding 96 well MaxiSorp flat bottom plate (Nunc, USA) and incubated over night at 4 °C. The plate was washed with 1xPBS three times and blocked with 2.5% BSA in 1xPBS for 1 hr at 37 °C. Following three washes with 1xPBST (0.2% Tween 20) and 1xPBS, the plate was incubated for 1 hr at 37 °C with 1:500 dilution of anti-rPfGDH and anti-peptides serum. The plate was then washed three time with 1xPBST and 1xPBS each. The bound anti-rPfGDH and anti-peptides were detected by goat-anti-mouse IgG-HRP antibodies (plate was washed after each step with 1xPBST and 1xPBS). Subsequently, the antigen-antibody reaction was developed with ELISA TMB substrate (KPL) for 20 min in dark at 37 °C and the reaction was stopped with 0.2 M H_2_SO_4_. The plate was read at 450 nm on Spectrostar ELISA plate reader. Animal sera without rPFGDH was used as negative control.

### Serum content from dried blood spot (DBS)

Three 3 mm discs from filter paper dried blood spots were punched out in 200μl of 1x phosphate buffered saline (PBS) containing 0.05% tween 20 and were incubated overnight at 37 °C. The following day, samples were centrifuged at 8000 g for 10 minutes and supernatant containing serum content was stored at −20 °C until further use.

### Detection of PfGDH in Clinical samples

In 96 well ELISA flat bottom plate, 10μl of clinical sample mixed with 1x sodium carbonate buffer (1:10) was coated in triplicate and incubated overnight at 4 °C. The following day, wells were washed with 1xPBST (GCC Biotech, India) and blocked with 2.5% BSA (Sigma) in 1xPBS (GCC Biotech) and incubated for 1 hr at 37 °C. The wells were washed thrice with 1xPBST and added with purified PfGDH antibodies in 1:1000 dilution and incubated for 3 hrs at 37 °C. Subsequently, wells were washed thrice as mentioned above. To detect PfGDH antibodies, goat-anti-mouse IgG-HRP antibodies added in wells in 1:5000 dilution and incubated for 45 min at 37 °C and wells were washed as described previously. In each well 100μl of TMB substrate (KPL) was added and incubated for 15 min in dark at 37 °C and 0.2 M H_2_SO_4_ was used to stop the reaction. The results were recorded at 450 nm on Spectrostar ELISA plate reader. Prism 8.0 was used to analyse and plot the data^21^ (Fig. 1).

## Conclusion

Preliminary results from quantitative ELISA using antibodies against PfGDH had shown strong affinity, sensitivity and specificity towards the GDH antigen from malaria patient. This is a novel work and was not reported by anyone on malaria cases from India. The results are promising to utilise GDH as a potential biomarker to detect *P. falciparum* in blood sample. However, present work will be further validated on large number and on different sample types including whole blood, serum, eluted DBS, saliva and urine from malaria patients infected with *P. falciparum* and other species.

## Supplementary information

Supplementary Information.
