# Supplementary material for: Glutamate dehydrogenase: a novel candidate to diagnose Plasmodium falciparum through rapid diagnostic test in blood specimen from fever patients
Source: Sci Rep. 2020 Apr 14;10:6307. doi: 10.1038/s41598-020-62850-x (PMC7156408; doi:10.1038/s41598-020-62850-x)
Supplement: Supplementary file 1 — Supplementary Information. [file 41598_2020_62850_MOESM1_ESM.pdf]

Glutamate dehydrogenase: a novel candidate to diagnose *Plasmodium falciparum* through rapid diagnostic test in blood specimen from fever patients

Lokesh D. Kori[a], Neena Valecha[b] and Anupkumar R. Anvikar[a]*

[a]ICMR-National Institute of Malaria Research, Dwarka-8, New Delhi- 110077

[b]Former Director, ICMR-National Institute of Malaria Research, Dwarka-8, New Delhi- 110077

*Corresponding author: anvikar@gmail.com

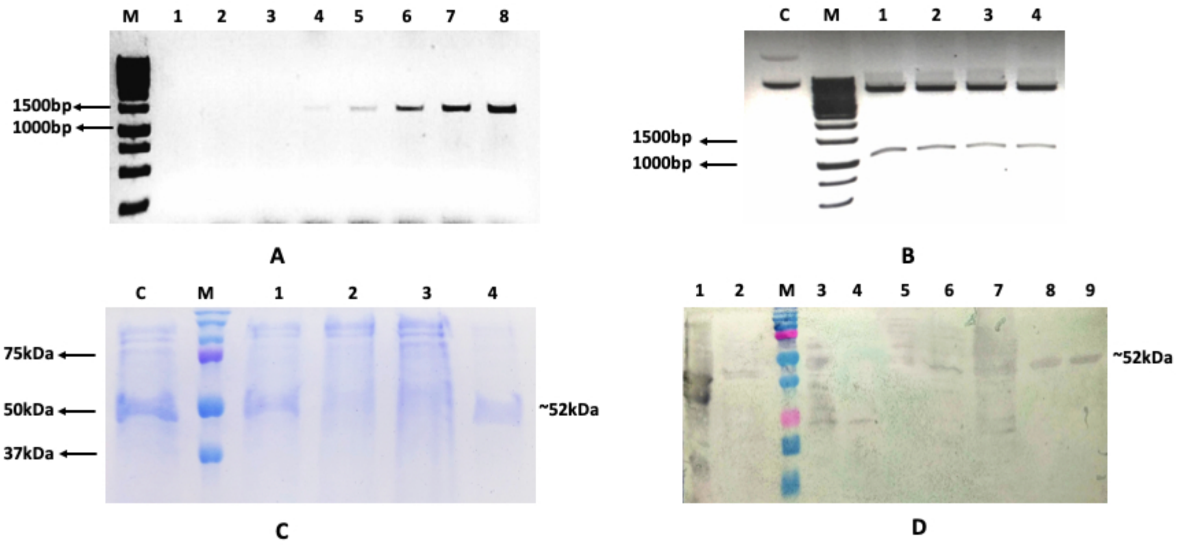

Figure 1.

A. Gene amplification, Lane M- 1Kb DNA ruler (Thermo scientific #SM0314), lane 1-8 rPfGDH gene amplification using gradient PCR at 70.0°, 68.9°, 67.0°, 64.0°, 60.7°, 57.9°, 56.1° and 55.0°C.

B. Restriction enzyme digestion, Lane – C intact control pET22b(+) vector, M - 1Kb DNA ruler, 1,2,3 and 4 are digested clone with rPfGDH gene (~1413bp).

C. Protein purification using Ni-IMAC gravity column, Lane – C rPfGDH cell lysate with 1M Guanidine hydrochloride, M- Dual colour protein Marker (Biorad), 1- rPfGDH cell lysate with 1M GuHCl, 2 - Flow through, 3- Wash with 20mM imidazole, 4- Elution with 50mM imidazole buffer.

D. Western Blot of rPfGDH, Lane 1 & 2- PfGDH cell lysate, M-Dual colour protein Marker (Biorad), 3 & 4 – flow through, 5 - wash with 20mM imidazole, 6 – rPfGDH flow through faction with 0.5M Guanidine HCl (GuHCl), 7 – PfGDH cell lysate with 2M GuHCl, 8 - rPfGDH sample with 20mM imidazole wash and 9 - elution with 50mM imidazole buffer.

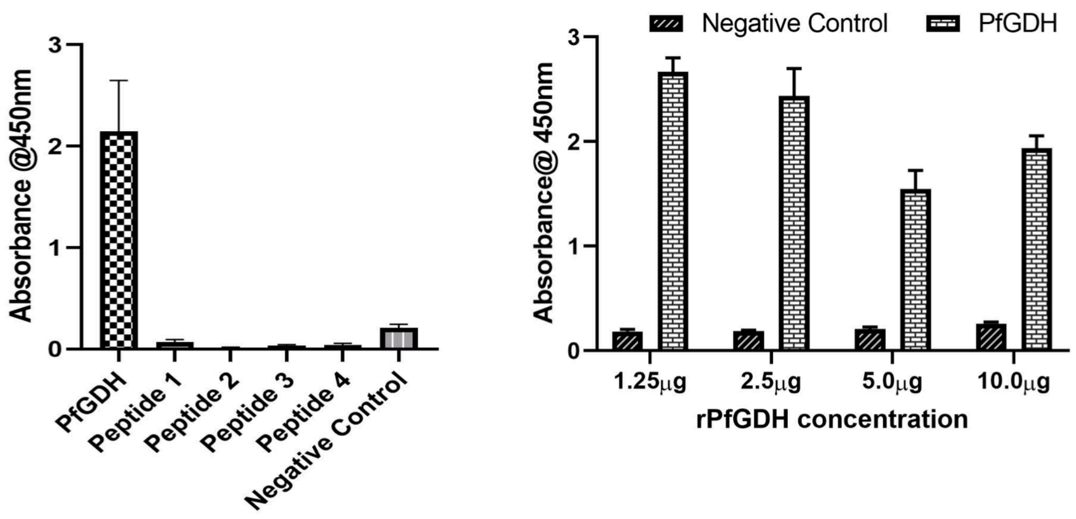

A **B**

Figure 2

A. Polyclonal antibodies response against the respective antigens (1μg) PfGDH, peptide 1-4 and negative control (serum from mice injected with 1x PBS).

B. ELISA results with various concentration of PfGDH and negative control (1xPBS).

Table 2. Comparison between study sample subgroups and P-values

	Infected RBC (14)	DBS (9)	Plasma (4)	Uninfected RBC (4)	Infected RBC vs DBS	Infected RBC vs Plasma	Infected RBC vs Uninfected RBC	DBS vs Plasma	DBS vs uninfected RBC	Plasma vs uninfected RBC
**Mean**	0.494	0.530	0.628	0.334	P = 0.378	P=0.018	P=0.005	P=0.092	P=0.002	P=0.000
**Std. Deviation**	0.098	0.064	0.160	0.010						
